# A model based on clinical data and multi-modal ultrasound for predicting cervical lymph node metastasis in patients with thyroid papillary carcinoma

**DOI:** 10.3389/fendo.2022.1063998

**Published:** 2022-12-12

**Authors:** Bin Wang, Qing Cao, Xin-Wu Cui, Christoph F. Dietrich, Ai-jiao Yi

**Affiliations:** ^1^ Department of Medical Ultrasound, Yueyang Central Hospital, Yueyang, China; ^2^ Department of Medical Ultrasound, Tongji Hospital, Tongji Medical College, Huazhong University of Science and Technology, Wuhan, China; ^3^ Department Allgemeine Innere Medizin, Kliniken Hirslanden Beau Site, Salem und Permanence, Bern, Switzerland

**Keywords:** contrast-enhanced ultrasound, shear wave elastography, thyroid papillary carcinoma, lymph node metastasis, predictive model

## Abstract

**Objective:**

The aim of this study was to explore diagnostic performance based on clinical characteristics, conventional ultrasound, Angio PLUS (AP), shear wave elastography (SWE), and contrast-enhanced ultrasound (CEUS) for the preoperative evaluation of cervical lymph node metastasis (CLNM) in patients with papillary thyroid carcinoma (PTC) and to find a reliable predictive model for evaluating CLNM.

**Materials and methods:**

A total of 206 thyroid nodules in 206 patients were included. AP, SWE, and CEUS were performed for all thyroid nodules. Univariate analysis and multivariate logistic regression analysis were performed to ascertain the independent risk factors. The sensitivity, specificity, and the area under the curve (AUC) of independent risk factors and the diagnostic model were compared.

**Results:**

Sex, age, nodule size, multifocality, contact extent with adjacent thyroid capsule, Emax, and capsule integrity at CEUS were independent risk predictors for CLNM in patients with PTC. A predictive model was established based on the following multivariate logistic regression: Logit (*p*) = −2.382 + 1.452 × Sex − 1.064 × Age + 1.338 × Size + 1.663 × multifocality + 1.606 × contact extent with adjacent thyroid capsule + 1.717 × Emax + 1.409 × capsule integrity at CEUS. The AUC of the predictive model was 0.887 (95% CI: 0.841–0.933), which was significantly higher than using independent risk predictors alone.

**Conclusion:**

Our study found that male presence, age < 45 years, size ≥ 10 mm, multifocality, contact extent with adjacent thyroid capsule > 25%, Emax ≥ 48.4, and interrupted capsule at CEUS were independent risk predictors for CLNM in patients with PTC. We developed a diagnostic model for predicting CLNM, which could be a potentially useful and accurate method for clinicians; it might be beneficial to surgical decision-making and patient management and for improving prognosis.

## Introduction

Papillary thyroid carcinoma (PTC) is the most common thyroid cancer, with 30%–80% of patients experiencing metastasis to the cervical lymph node (1). Cervical lymph node metastasis (CLNM) can affect surgical options and extent, and it is related to thyroid cancer recurrence and patients’ prognosis (2, 3). Therefore, accurate preoperative evaluation of CLNM is extremely important.

Ultrasound is the first-line imaging modality for preoperative CLNM assessment in patients with suspicious malignant nodules (2). Preoperative ultrasound can find suspicious cervical lymphadenopathy in 20%–31% of patients, which might potentially alter the surgical approach (2, 4, 5). However, ultrasound has a low sensitivity in the diagnosis of central lymph node metastasis (10.5%–61%) (6), and metastases are most likely to occur in central lymph nodes. Therefore, it is important to find a method to accurately assess CLNM.

The Angio PLUS microvascular Doppler ultrasound technique (AP) is a novel Doppler technique in the supersonic imaging system. It has been used in other organs such as breast or parathyroid (7, 8) and could detect more low-speed microvessels compared with color Doppler flow imaging. Thus, we expected AP to provide more microvessel information for predicting CLNM.

Shear wave elastography (SWE) can quantitatively assess tissue hardness by obtaining Young’s modulus, along with a color-coded elasticity map. Recently, SWE has been widely used in the diagnosis of thyroid nodules, which can be a potential indicator in the diagnosis of thyroid nodules and provide additional information for clinical decision-making (9). However, there were a few studies (6, 10) on the clinical utility of quantitative SWE in predicting CLNM, which found that a high index of thyroid nodules was independently related to CLNM (6), but the optimal cutoff values of Young’s modulus were controversial; therefore, it is insufficient to use SWE alone to evaluate CLNM.

Contrast-enhanced ultrasound (CEUS) can present the macro- and micro-vascularization of tumor compared with the surrounding tissues. Recently, CEUS has been widely used in differentiating between benign and malignant thyroid nodules and is a promising noninvasive method in the diagnosis of thyroid nodules (11). Several studies have reported that CEUS is useful for the evaluation of biological behavior and CLNM (12–14), but the enhancement patterns in different studies were inconsistent; thus, it is important to conduct further studies.

CLNM was difficult to evaluate before surgery, especially for central lymph nodes. Clinical characteristics, conventional ultrasound, AP, SWE, and CEUS could provide information for thyroid nodules, which may be useful for the preoperative evaluation of CLNM. To the best of our knowledge, combining the use of clinical characteristics, conventional ultrasound, AP, SWE, and CEUS for the preoperative evaluation of CLNM has rarely been reported. The purpose of this study was to explore the diagnostic performance based on clinical characteristics, conventional ultrasound, AP, SWE, and CEUS for the preoperative evaluation of CLNM and to find a reliable predictive model for CLNM, which would be beneficial to surgical decision-making and for improving patient prognosis.

## Materials and methods

This prospective study was approved by the ethics committee of Yueyang Central Hospital, and all patients signed informed consent before CEUS examination and surgery.

### Patients

From March 2020 to May 2022, a total of 582 patients with 582 thyroid nodules were evaluated initially. Of these, 206 thyroid nodules in 206 patients were finally enrolled. The inclusion criteria were as follows: (a) the pathology of thyroid nodule in each patient was confirmed as PTC *via* surgery, (b) surgery was conducted within 1 month after SWE and CEUS examination, and (c) age 18 years or older. The exclusion criteria were as follows: (a) had a previous needle biopsy, (b) had previous radiofrequency ablation (RFA), and (c) had a contraindication of CEUS: history of hypersensitivity reactions to sulfur hexafluoride (SonoVue^®^, Bracco International, Milan, Italy) or to any of the inactive ingredients in SonoVue.

### Ultrasound examination

Conventional ultrasound, AP, SWE, and CEUS examinations were conducted using an Aixplorer ultrasound system (Supersonic imaging, France) equipped with an L15-4 linear array transducer for conventional ultrasound or AP and an L10-5 linear array transducer for SWE or CEUS by the same investigator.

Conventional ultrasound examination: The conventional ultrasound was performed with an L15-4 linear array transducer, and the conventional ultrasound images were obtained by scanning the suspicious target thyroid nodules and all regions of cervical lymph nodes, and then the general characteristics were recorded, including location, size, multifocality, composition, echogenicity, shape, margin, echogenic foci (15), and the extent of contact between thyroid nodules and adjacent thyroid capsule (16). All suspicious thyroid nodules were classified as ACR TI-RADS 3 (mildly suspicious), ACR TI-RADS 4 (moderately suspicious), or ACR TI-RADS 5 (highly suspicious) (15), and these nodules were recommended for CEUS and SWE examinations. If there was more than one suspicious thyroid nodule, we chose the highest suspicious thyroid nodule. Multifocality is defined as more than one suspicious thyroid nodule in conventional ultrasound and more than one confirmed malignant nodule by surgical pathology.

AP examination: AP vascularization of nodules was classified into three grades (17): Grade 0, no blood flow in the nodule; Grade I: a few spots of blood flow or one long vessel penetrating into the nodule; Grade II: abundant blood flow with five or more punctate blood flows or two long vessels inside nodules.

SWE examination: After AP examination, the system was changed to SWE mode with an L10-5 linear array transducer. In general, to decrease the effect of artery pulsation on SWE measurement, longitudinal section is usually selected to conduct SWE imaging. Patients should hold their breath for several seconds while the SWE was conducted. The stiffness range of the color map was from blue to red (0–180 kPa). The elasticity characteristics were measured using the quantification box (Q-box), which should contain the whole nodules, excluding the surrounding tissues, and then the system automatically calculated elasticity parameters, including Emin, Emean, and Emax. The median of five measurements was taken.

CEUS examination: After SWE examination, CEUS was performed with the same linear array transducer. The patients should breathe quietly without swallowing, coughing, and talking. To display suspicious target nodules clearly, the double-contrast mode was used. Sulfur hexafluoride was used in this study. A total of 25 mg of sulfur hexafluoride, diluted in 5 ml of 0.9% sodium chloride, was administered to each patient as a 2.4-ml intravenous bolus, followed by a 5-ml saline flush. Then, CEUS imaging was continuously recorded for 90 s in the machine’s hard disk.

According to previous studies (14, 18, 19) and our clinical experience, the enhancement patterns of thyroid nodules were evaluated with the following features: peak enhancement intensity (hyper-enhancement, iso-enhancement, or hypo-enhancement); ring enhancement was classified as present or absent; homogeneity of enhancement (homogeneous or heterogeneous); contrast agent arrival time (synchronous with, earlier than, or later than adjacent thyroid tissue); enhancement direction (scattered, centripetal, or centrifugal); enhancement area (equal to, greater than, or less than that on conventional ultrasound); nodule composition at CEUS (nonsolid or solid); enhancement border (the border between the nodule and the surrounding parenchyma at the peak intensity) was classified as well-defined or ill-defined; and capsule integrity at CEUS (the membrane was defined as continuous if the thyroid capsule showed a line-like intact structure) was classified as continuous and interrupted.

Imaging analysis: The same investigator, who had more than 5 years of experience in thyroid conventional ultrasound and 3 years of experience in AP, SWE, and CEUS, carried out all ultrasound examinations. General clinical data, conventional ultrasound, AP, SWE, and CEUS imaging were recorded. According to the pathology after surgery, enrolled patients were divided into the cervical lymph node non-metastasis group and the metastasis group.

### Statistical analysis

SPSS 23.0 and MedCalc 19.0 were used for all statistical analysis. A receiver operating characteristic (ROC) curve differentiating the non-metastasis group from the metastasis group was drawn based on Young’s modulus for each nodule. The optimal cutoff value and area under the curve (AUC) were calculated. The count data of clinical characteristics, AP, SWE, and the enhancement patterns of CEUS were compared with Kappa analysis or Fisher’s exact test. *p* < 0.05 was regarded as the threshold for statistical significance. In order to identify the independent risk factors, variables that show univariate significance for CLNM were added to a multivariate logistic regression analysis. The diagnostic performance of independent risk factors and the diagnostic model was calculated with ROC, and the sensitivity, specificity, and the AUC of independent risk factors and the diagnostic model were compared.

## Results

### General clinical data and conventional ultrasound features between the non-metastasis group and the metastasis group

**Figure 1:**
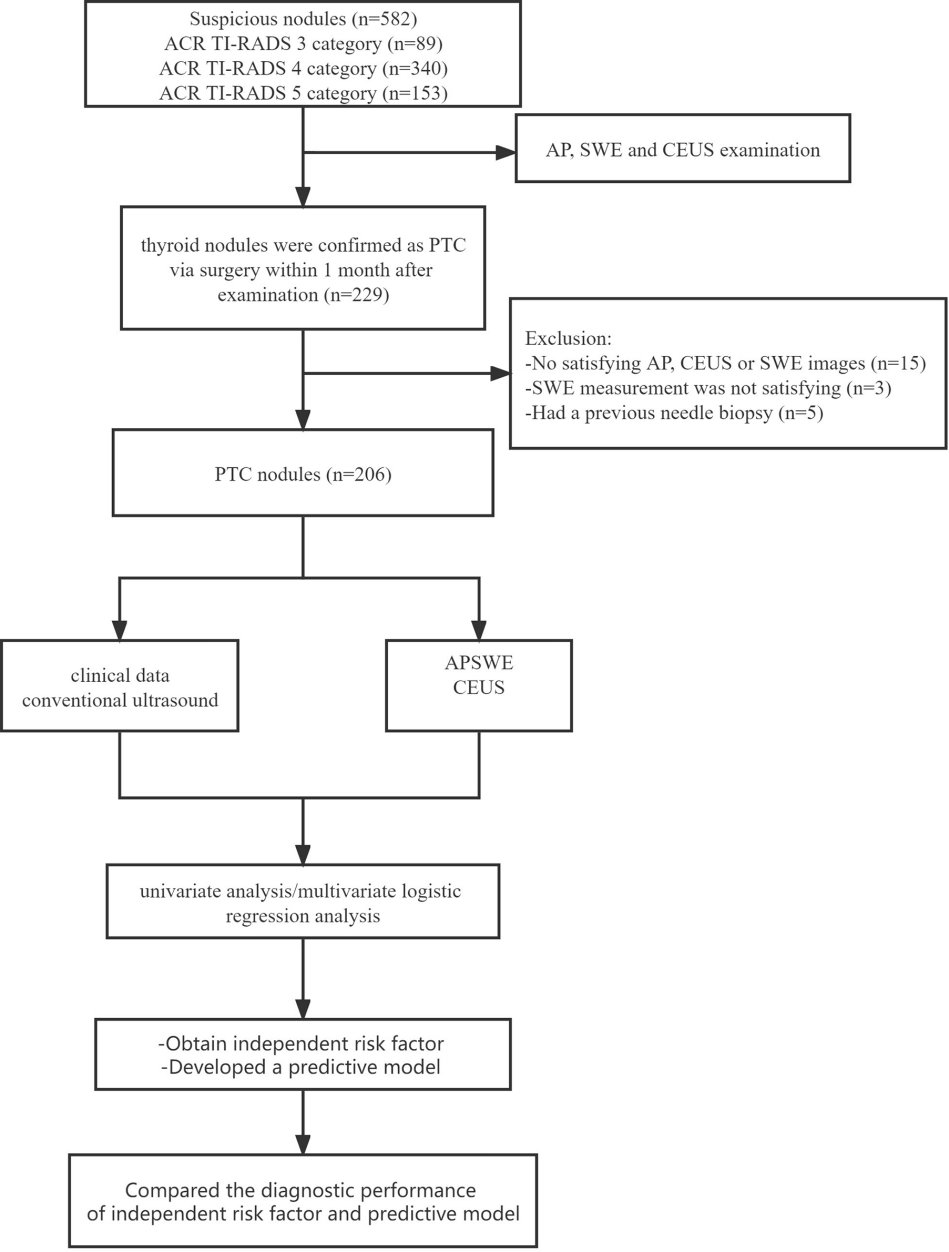
The flowchart of selection of thyroid carcinoma patients.

We chose the highest suspicious thyroid nodule for patients with more than one suspicious thyroid nodule, which was confirmed PTC by surgical pathology. Neck dissection was performed in all 206 enrolled patients. In the end, the surgically pathological results showed that 123 nodules were PTC without CLNM and 83 nodules were PTC with CLNM (**Figure 1**). The rate of CLNM was 40.29% in this study. All the nodules were classified into either the non-metastasis group or the metastasis group. General clinical characteristics and conventional ultrasound features are summarized in **Table 1**. Sex, age, size, multifocality, margin, calcification, and contact extent with adjacent thyroid capsule were statistically different between the non-metastasis group and the metastasis group.

**Table 1:** Clinical data and conventional ultrasound features between the non-metastasis and metastasis group.

Conventional and clinical features		Non-metastatic group	Metastatic group	*χ*2	p-value
		*n* = 123	*n* = 83		
Sex	Female	107	59	8.015	0.005
	Male	16	24		
Age	<45	31	36	7.456	0.006
	≥45	92	47		
Location	Upper	21	20	1.549	0.461
	Middle	54	32		
	Lower	43	29		
Multifocality	No	98	47	12.631	0.000
	Yes	25	36		
Composition	Mixed cystic and solid	2	2	0.16	0.689
	Solid or almost completely solid	121	81		
Echogenicity	Isoechoic/hyperechoic	5	4	0.067	0.795
	Hypoechoic/very hypoechoic	118	79		
Shape	Wider than tall	19	21	3.705	0.079
	Taller than wide	104	62		
Margin	Smooth	67	36	5.274	0.022
	Ill-defined/lobulated or irregular	56	57		
Calcification	None or large comet-tail artifacts	53	14	15.527	0.000
	Macrocalcifications or peripheral calcifications or punctate echogenic foci	70	69		
Size	<10 mm	109	35	50.822	0.000
	≥10 mm	14	48		
Contract with the adjacent thyroid capsule	≤25%	86	15	52.763	0.000
	>25%	37	68		
HT	No	77	52	0.000	0.994
	Yes	46	31		

### AP, SWE, and CEUS features between the non-metastasis group and the metastasis group

AP, SWE, and CEUS features between the non-metastasis group and the metastasis group are summarized in **Table 2**. AP vascularization was statistically different between the non-metastasis group and the metastasis group. The CLNM rate of Grade 0–I and Grade II were 34.5% and 52.2%, respectively.

**Table 2:** AP, SWE, and CEUS features between the non-metastasis and metastasis group.

		Non-metastatic group	Metastatic group	*χ* ^2^	*p*-value
		*n* = 123	*n* = 83		
AP	0	12	9	6.604	0.037
	I	79	39		
	II	32	35		
SWE	Emin <26.45	84	42	6.529	0.011
	Emin ≥26.45	39	41		
	Emean <36.05	80	32	14.014	0.000
	Emean ≥36.05	43	51		
	Emax <48.4	84	26	27.216	0.000
	Emax ≥48.4	39	57		
CEUS					
Ring enhancement	Present	2	3	—	0.394
	Absent	121	80		
Peak intensity	Hyper-enhancement/iso-enhancement	19	9	0.894	0.344
	Hypo-enhancement	104	74		
Homogeneity	Homogeneous	18	5	3.704	0.054
	Heterogeneous	105	78		
Enhancement direction	Scattered	15	11	0.050	0.823
	Centripetal or centrifugal	108	72		
Contrast agent arrival time	Synchronous with normal thyroid tissue	28	19	0.000	0.983
	Earlier or later than normal thyroid tissue	95	64		
Enhancement area	Equal to conventional ultrasound	62	41	0.020	0.887
	Smaller or greater than conventional ultrasound	61	42		
Nodule composition at CEUS	Nonsolid	1	2	—	0.566
	Solid	122	81		
Capsule integrity at CEUS	Continuous	104	27	57.93	0.000
	Interrupted	19	56		
Enhancement border	Clear	48	55	14.71	0.000
	Unclear	75	28		

An ROC curve was drawn based on Emin, Emean, and Emax to calculate the optimal cutoff value for discriminating the non-metastasis from the metastasis group. The optimal cutoff value was 26.45 kPa for Emin, 36.05 kPa for Emean, and 48.4 kPa for Emax.

There were significant differences in capsule integrity at CEUS and enhancement border at CEUS between the non-metastasis group and the metastasis group. There were no significant differences in peak enhancement intensity, ring enhancement, homogeneity of enhancement, contrast agent arrival time, enhancement direction, enhancement area, and nodule composition at CEUS.

### Univariate and multivariate analysis on the predictors of cervical lymph node metastasis

A summary of the binary logistic regression analysis of clinical data, conventional ultrasound, AP, SWE, and CEUS features is shown in **Table 3**. Sex, age, nodule size, multifocality, contract extent with adjacent capsule, Emax, and capsule integrity at CEUS were independent risk predictors for CLNM in patients with PTC. A predictive model was established according to the multivariate logistic regression: Logit (*p*) = −2.382 + 1.452 × Sex − 1.064 × Age + 1.338 × Size + 1.663 × multi-focality + 1.606 × contact extent with adjacent thyroid capsule + 1.717 × Emax + 1.409 × capsule integrity at CEUS.

**Table 3:** Multivariate logistic regression analysis of clinical data, conventional ultrasound, AP, SWE, and CEUS.

Variables	*B*	*p*	OR	95% CI for OR	
Sex	1.452	0.010	4.270	1.423	12.817
Age	−1.064	0.023	0.345	0.138	0.866
Size	1.338	0.020	3.811	1.229	11.814
Multifocality	1.663	0.001	5.274	2.014	13.809
Margin	0.715	0.121	2.044	0.827	5.050
Calcification	0.698	0.192	2.009	0.704	5.735
Contact extent	1.606	0.001	4.980	2.009	12.346
AP	−0.797	0.060	0.451	0.196	1.034
Emin	−0.169	0.797	0.845	0.234	3.056
Emean	−1.150	0.184	0.317	0.058	1.730
Emax	1.717	0.021	5.570	1.296	23.942
CEUS border at CEUS	−0.398	0.397	0.672	0.268	1.685
Capsule integrity at CEUS	1.409	0.004	4.092	1.583	10.576
Constant	−2.328	0.002	0.097		

### Comparing the diagnostic performance of independent risk predictors and the predictive model

**Figure 2:**
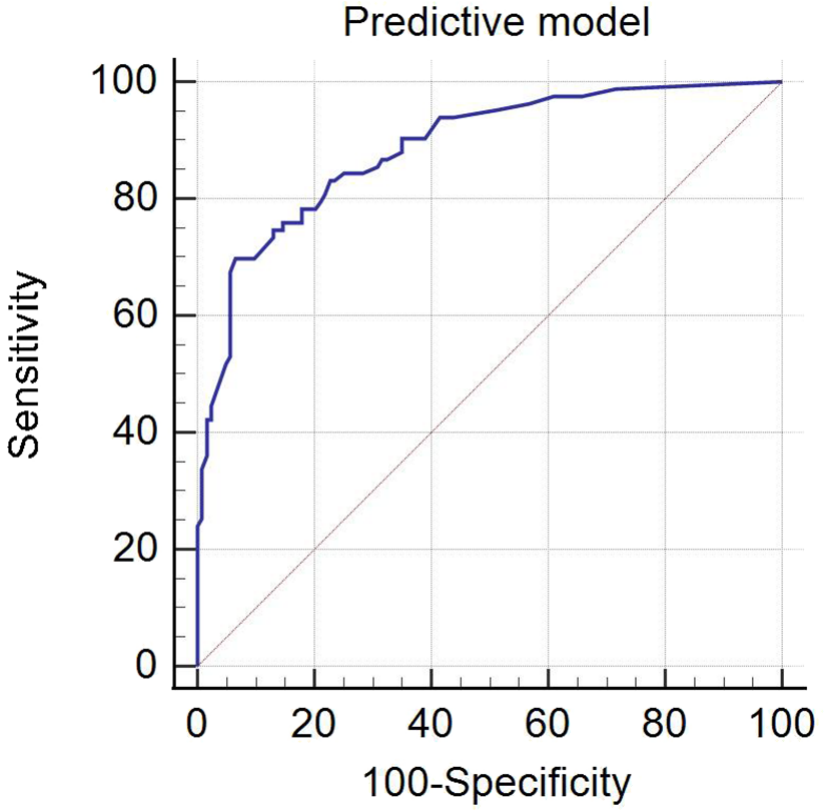
The ROC of the predictive model.

ROC analysis of the predictive model for evaluating CLNM was performed (**Figure 2**). The AUC of the predictive model was 0.887 (95% CI: 0.841–0.933), which was significantly higher than using independent risk predictor alone (**Table 4**). The predictive model also had the best sensitivity and specificity, compared with using independent risk factor alone; the sensitivity and specificity of the predictive model were 69.88% and 93.50%, respectively.

**Table 4:** The AUC of independent risk factors and predictive model.

	Sensitivity	Specificity	AUC	95% Confidence Interval
Sex	28.90%	87.00%	0.580	0.499–0.661
Age	43.40%	74.80%	0.591	0.511–0.671
Size	57.80%	88.60%	0.732	0.658–0.806
Multifocality	43.45%	79.70%	0.615	0.536–0.695
Contact extent with adjacent thyroid capsule	68.70%	54.50%	0.685	0.610–0.760
Emax	68.70%	68.30%	0.685	0.610–0.760
Capsule integrity at CEUS	67.50%	84.60%	0.760	0.690–0.831
Predictive model	69.88%	93.50%	0.887	0.841–0.933

## Discussion

In this study, we analyzed clinical data, conventional ultrasound feature, AP, SWE, and CEUS enhancement patterns of thyroid nodules in PTC patients to explore their predictive values for CLNM. We found that male presence, age < 45 years, size ≥ 10 mm, multifocality, contact extent with adjacent thyroid capsule > 25%, Emax ≥ 48.4, and interrupted capsule at CEUS were independent risk predictors for CLNM in patients with PTC. A predictive model based on multivariate logistic regression analysis showed better diagnostic performance for CLNM with an AUC of 0.887, compared with using independent risk predictor alone.

Several studies (14, 20) had reported that clinical data of patients including sex or age had predictive values for the aggressiveness of PTC. In this study, we found male and age < 45 years were independent risk factors for predicting CLNM in patients with PTC, which was consistent with previous studies. The CLNM rate of female patients (35.5%) was significantly lower than that of male patients (60.0%) in this study, and the CLNM rate of patients ≥ 45 years (35.5%) was lower than that of patients < 45 years (50%). Thus, one should practice caution in the surgery decision-making regarding and the preoperative evaluation of young male patients.

The size of malignant thyroid nodules ≥10 mm was an independent risk factor for predicting CLNM in patients with PTC, which was consistent with previous studies (21, 22). In general, the characteristics of thyroid nodules might be determined already during the initial formation, and the size of nodules was related to uncontrolled cell division and proliferation. Thus, the larger the PTC nodule was, the faster it grows and the higher the degree of nodule infiltration; hence, it is more prone to CLNM.

Multifocality presents a reported incidence of about 18%–27% in patients with PTC (22), which is related to lymph node metastasis, recurrence, and prognosis (23). In this study, we defined multifocality as more than one suspicious thyroid nodule in conventional ultrasound and more than one confirmed malignant nodule by surgical pathology. However, microscopic PTC is still difficult to detect prior to surgery. In this study, multifocality was an independent risk factor for predicting CLNM in PTC patients (*p* = 0.001), which is consistent with a previous study. The OR value of multifocality (OR = 5.274) in this study was significantly higher than previous studies (OR = 1.297–3.235) (14, 18, 24).

The ill-defined, lobulated, or irregular margin of thyroid nodules is generally associated with the irregular growth of the fibrous stroma surrounding the PTC nodules or carcinoma invasion of the surrounding thyroid parenchyma. However, margin was not an independent risk factor for predicting CLNM in patients with PTC in this study, which was consistent with most previous studies (14, 18, 22).

Whether calcification can predict CLNM of PTC is still controversial; some studies (22, 25) reported that micro-calcification could reflect the rapid growth in carcinoma tissues, and ≥5-mm micro-calcifications was an independent risk factor for predicting CLNM in patients with PTC, while several studies (12, 14) did not have the same results. In this study, we found that calcifications were significantly different between the non-metastasis and metastasis group, but it was not an independent risk factor for predicting CLNM according to multivariate logistic regression analysis. Thus, the correlation between calcifications and CLNM in PTC needs to be explored in a multi-center large-sample study in the future.

Previous studies (16) have found that contact extent with adjacent thyroid capsule >25% was the most accurate predictive feature for extra-thyroidal extension in this study, and we found that contact extent with adjacent thyroid capsule >25% was an important independent factor for CLNM. It may be because extra-thyroidal extension breaks the thyroid capsule, and the thyroid gland has a rich lymphatic network, which could easily cause CLNM.

HT is a common autoimmune thyroid disease, and the correlation between HT and PTC has been widely debated and remains controversial (26). Moreover, the enlarged lymph node in the central region caused by thyroiditis is always easily confused with lymph node metastasis. Li (10) found that HT has no significant difference for predicting CLNM in patients with PTC, which is consistent with our study.

A previous study reported that the characteristics of superb microvascular imaging (SMI) in PTC nodules (Grade II) were independent risk predictors for CLNM in patients with PTC, suggesting that the degree of rich and disorderly blood flow in PTC nodules might be helpful to predict CLNM (22). In this study, we found that AP vascularization in PTC nodules was significantly different between the non-metastasis and metastasis group, which was consistent with a previous study (22); the CLNM rate of Grade 0–I and Grade II was 34.5% and 52.2%, respectively. However, we did not obtain the same result by multivariate logistic regression analysis, and the *p*-value (0.06) was slightly higher than 0.05, which could be affected by the sample size.

SWE has been widely used to quantitatively assess the thyroid nodules. Few studies (6, 10, 27) have explored the values of SWE for predicting CLNM in patients with PTC. Park et al. (6) found that higher elasticity values of PTC were related to CLNM. Another study (27) found that the elasticity characteristics of PTC nodules did not have a significant difference between the non-metastasis and metastasis group. Li et al. (10) reported that Emax was an independent risk factor for CLNM, and the best cutoff value of Emax was >59.0 kPa. Similarly, Emax was an independent risk factor for predicting CLNM based on multivariate logistic regression analysis in this study, which was consistent with a previous study; using Emax > 48.4 kPa, our OR (OR = 5.57) was significantly higher than the OR of previous studies (OR = 1.005–4.934) (6, 10). Thus, the higher the Emax was, the more careful we should be in clinical work.

Several studies have reported that the enhancement patterns of CEUS were useful for predicting CLNM. Zhan et al. (12) found that peak enhancement intensity and homogeneity were significant features in predicting CLNM in PTC patients. Hong et al. (28) reported that hyper-enhancement or iso-enhancement could be an independent risk factor for predicting CLNM in PTC patients. Xue et al. (14) found that hyper-enhancement was not an independent predictor for predicting CLNM. In this study, we found that there was no statistical difference on peak enhancement intensity, ring enhancement, homogeneity of enhancement, contrast agent arrival time, enhancement direction, enhancement area, and nodule composition at CEUS between the non-metastasis and metastasis group, which was different with the previous studies; it may be because the qualitative enhancement patterns of CEUS could be controversial in different observers and ultrasound systems. The interrupted capsule at CEUS was an independent risk factor for predicting CLNM in patients with PTC, which is related to extra-thyroidal extension. Thus, the rate could be higher due to the rich lymphatic network in the gland network. When using the interrupted capsule at CEUS alone for predicting CLNM, the specificity was 84.60%, which showed that the rate of CLNM was low with the continuous capsule at CEUS.

**Figure 3:**
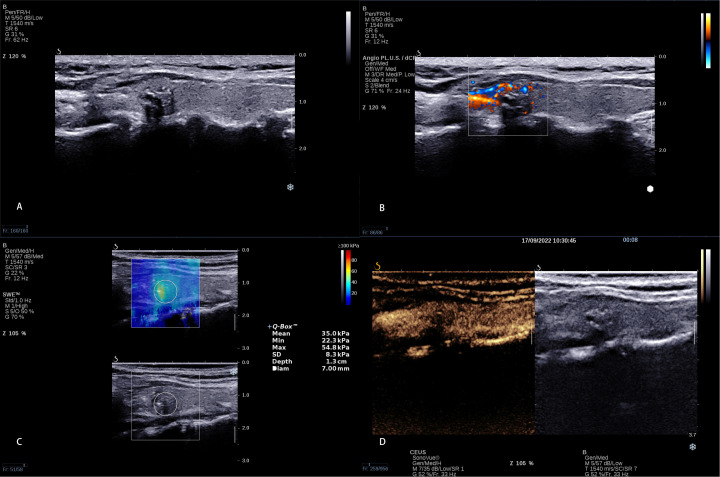
A 8 × 6 mm thyroid nodule in the left lobe of a 38-year-old woman. **(A)** Conventional ultrasound showed a solid hypo-echoic, irregular nodule with calcifications; the shape was wider than taller and the contact extent with adjacent thyroid capsule >25%. **(B)** AP shows that the vascularization of nodule could be classified as Grade I **(C)** SWE measurement shows that Emin, Emean, and Emax were 22.3 kPa, 35.0 kPa, and 54.8 kPa, respectively. **(D)** CEUS shows interrupted capsule at CEUS. The calculated predictive value with the logistic regression formula was 2.35 (>2.0805), which was regarded as presence of CLNM.

**Figure 4:**
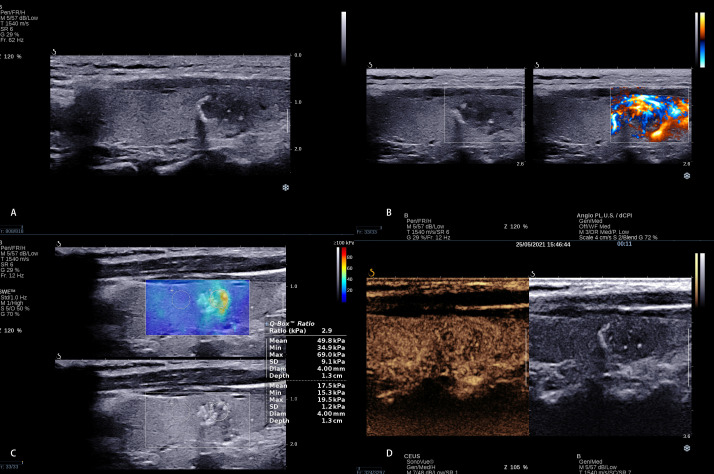
A 13 × 7 mm thyroid nodule in the right lobe of a 31-year-old woman. **(A)** Conventional ultrasound showed a solid hypo-echoic, ill-defined nodule with calcifications; the shape was wider than taller and the contact extent with adjacent thyroid capsule <25%. **(B)** AP shows that the vascularization of nodule could be classified as Grade II. **(C)** SWE measurement shows that Emin, Emean, and Emax were 34.9 kPa, 49.8 kPa, and 69.0 kPa, respectively. **(D)** CEUS shows continuous capsule at CEUS. The calculated predictive value with the logistic regression formula was 0.673 (<2.0805), which predicted no CLNM.

Based on the multivariate logistic regression analysis of clinical characteristics, conventional ultrasound, AP, SWE, and CEUS, we developed a predictive model for predicting CLNM in PTC patients (**Figures 3**, **4**); the model had the best AUC (0.887, 95% CI: 0.841–0.933) compared with using the independent factor alone. Moreover, this predictive model in this study was simple, convenient, and accurate, which could be widely used in clinical work.

There are several limitations in this study. First, it was a single-center study with a small sample. Second, quantitative CEUS features were not included in this study. Third, we did not compare inter-observer and intra-observer variation.

## Conclusion

Based on the multivariate logistic regression analysis of clinical characteristics, conventional ultrasound, SWE, and CEUS, we found that male presence, age < 45 years, size ≥ 10 mm, multifocality, contact extent with adjacent thyroid capsule > 25%, Emax ≥ 48.4, and interrupted capsule at CEUS were independent risk predictors for CLNM in patients with PTC. We developed a diagnostic model for predicting CLNM, which could be a potentially useful and accurate method for clinicians; it would be beneficial to surgical decision-making and patient management and for improving prognosis.

## Data availability statement

The raw data supporting the conclusions of this article will be made available by the authors, without undue reservation.

## Ethics statement

The studies involving human participants were reviewed and approved by the ethics committee of Yueyang Central Hospital. The patients/participants provided their written informed consent to participate in this study.

## Author contributions

Conception and design: BW, A-jY, X-WC, and CD. Drafting of the article: BW, QC, and A-jY. Critical revision of the article for important intellectual content: BW, QC, and X-WC. All authors contributed to the article and approved the submitted version.
